# Ballads of Old Doctors

**Published:** 1906-02

**Authors:** 


### BALLADE OF OLD DOCTORS.

Where are the doctors of old time;
  Their methods, systems, treatises?
In modern practice, 'twere a crime,
  To utilize antiques like these.
The things that to Hippocrates
  Appeared so eminently just
According to his theories,
  Have mingled with the centuries' dust.

Where is the German quack sublime?
  Thy potions, pills and alchemies,
O Paracelsus Hohenheim!
  Despite thy loud assurances,
Are rank and stale as mouldy cheese
  And in thy vaunted metals rust
Has bit: these with thy crack-brained fallacies
  Have mingled with the centuries' dust.

New years ring in with merry chime.
  Time laughs at his own vagaries
And covers with a frosty rime
  Man's foibles, fads, inanities.
Still, as of yore, parades disease—
  The doctor into earth is thrust—
Stalks onward when its enemies
  Have mingled with the centuries' dust.

L' ENVOI.

Wilt thou, O Time! in thy decrees
  Likewise *our* boastful claims adjust,
When *modern* doctors, patients, fees
  Have mingled with the centuries' dust?

—B. W.

---

A YOUNG woman, newly married and in the family way, was speaking to some of her friends and remarked that she was very fortunate in the selection of her doctor and nurse, and especially the latter, as she usually refused to accept *stereopticon* cases. Her husband also stated that his wife could go out without causing much comment, as she wore *maturity* gowns. He meant to say maternity gowns.

